# X-Linked Lymphoproliferative Disease Type 1: A Clinical and Molecular Perspective

**DOI:** 10.3389/fimmu.2018.00666

**Published:** 2018-04-04

**Authors:** Neelam Panchal, Claire Booth, Jennifer L. Cannons, Pamela L. Schwartzberg

**Affiliations:** ^1^Molecular and Cellular Immunology Section, Great Ormond Street Institute of Child Health, University College London, London, United Kingdom; ^2^Department of Pediatric Immunology, Great Ormond Street Hospital for Children NHS Foundation Trust, London, United Kingdom; ^3^National Human Genome Research Institute, National Institutes of Health, Bethesda, MD, United States; ^4^National Institute of Allergy and Infectious Diseases, National Institutes of Health, Bethesda, MD, United States

**Keywords:** X-linked lymphoproliferative disease 1, Epstein–Barr virus, SAP (signaling lymphocyte activation molecule-associated protein), signaling lymphocytic activation molecule, primary immunodeficiency disease, hemophagocytic lymphohistiocytosis, hematopoietic stem cell transfer, gene therapy

## Abstract

X-linked lymphoproliferative disease (XLP) was first described in the 1970s as a fatal lymphoproliferative syndrome associated with infection with Epstein–Barr virus (EBV). Features include hemophagocytic lymphohistiocytosis (HLH), lymphomas, and dysgammaglobulinemias. Molecular cloning of the causative gene, *SH2D1A*, has provided insight into the nature of disease, as well as helped characterize multiple features of normal immune cell function. Although XLP type 1 (XLP1) provides an example of a primary immunodeficiency in which patients have problems clearing primarily one infectious agent, it is clear that XLP1 is also a disease of severe immune dysregulation, even independent of EBV infection. Here, we describe clinical features of XLP1, how molecular and biological studies of the gene product, SAP, and the associated signaling lymphocyte activation molecule family receptors have provided insight into disease pathogenesis including specific immune cell defects, and current therapeutic approaches including the potential use of gene therapy. Together, these studies have helped change the outcome of this once almost uniformly fatal disease.

## Introduction

Epstein–Barr virus (EBV) is a highly prevalent human gamma herpes virus that is spread *via* saliva and primarily infects the oropharyngeal epithelium and B cells (1). Infection in children is usually very mild, whereas in teenager and adults, it can result in a picture of infectious mononucleosis (IM) with fevers, flu-like symptoms, and even systemic lymphoproliferative disease. Studies suggest that EBV has infected approximately 90% of adults. After infection, EBV remains latent in B cells for the remainder of the life of the host, and although most people remain asymptomatic, it can cause B cell and T cell lymphomas, Hodgkin lymphoma, and Burkitt’s lymphoma in certain groups, particularly in immunocompromised patients (2).

A major and critical issue with EBV arises in the case of such immunocompromised individuals, including those presenting with monogenic deficiencies, where EBV infection leads to a wide range of clinical complications and acquired disease phenotypes (3). In this review article, we will explore the disease pathologies arising in patients with a rare form of primary immunodeficiency (PID), X-linked lymphoproliferative disease type 1 (XLP1), which is perhaps the classic example of a PID associated with an inability to clear EBV (3–5).

## EBV in XLP Disease

### Characterization of Early Cases

X-linked lymphoproliferative disease or Duncan’s disease was described in the mid-1970s by Purtilo and colleagues in the Duncan kindred, where 6 out of 18 young males died of a lymphoproliferative disorder (6, 7). Three of these males had IM either immediately prior to or concurrent with the development of disease, which included fatal IM, hemophagocytic syndrome, and B cell malignancies, as well as humoral immune defects such as dysgammaglobulinemia. The observation of EBV-driven manifestations associated with a primary immune-deficiency catalyzed the recognition of XLP. Of note, two other contemporary reports also described families with males who succumbed to lymphoproliferative disorders and/or agammaglobulinemia associated with EBV infection, who may have had the same syndrome (8, 9).

Early investigations carried out by Purtilo and his team aimed to understand why EBV infection led to such aggressive and often fatal clinical phenotypes in these patients. In 1980, an XLP registry was established (7), which tracked presumed XLP patients with regard to disease onset and progression. The study revealed that the majority of patients had succumbed to IM due to extensive liver pathology and lymphoid infiltration of organs. However, those who did survive, as well as some EBV-negative male relatives, still progressed to develop dysgammaglobulinemia and/or B cell malignancies (10, 11). By 1995, over 270 boys were registered from over 80 kindreds (12); the overall mortality was reported as 75% with the majority of boys dying before 10 years of age, proving the severity of this condition.

The cloning of the gene, *SH2D1A*, responsible for this disease both revealed phenotypes in family members before they presented with the classic picture of EBV-induced pathology and allowed further molecular understanding of what is now called XLP type 1 (XLP1) (13–15). Clinical manifestations of XLP1 are now recognized to include a wider range of phenotypes associated with immune dysregulation even independent of EBV infection (5, 16). It should be noted that mutations in a second gene, *BIRC4*, encoding the X-linked inhibitor of apoptosis, XIAP, have been described in a subset of XLP patients who did not carry mutations in *SH2D1A* (now referred to as having XLP2) (17). However, XLP2 is more closely associated with EBV-driven hemophagocytic lymphohistiocytosis (HLH), as well as other clinical features not found consistently in XLP1 such as splenomegaly and colitis and will not be discussed further in this review (17, 18).

### Clinical Features

The main clinical features of XLP1 remain HLH, dysgammaglobulinemia, and lymphoma but other described manifestations include aplastic anemia, vasculitis, chronic gastritis, and skin lesions (12, 19–24). HLH is the most common and lethal presentation, tending to occur early in childhood and associated with significant mortality, with a proportion of patients succumbing before hematopoietic stem cell transplant (HSCT) (16). HLH is a multisystem syndrome caused by hyperinflammation resulting in immune dysregulation, tissue damage, and often multiorgan failure. The main features are fever, cytopenias, and hepatosplenomegaly but involvement of other organs is often seen. Diagnostic criteria are available (25).

Up to 50% of patients demonstrate a range of humoral immune abnormalities, ranging from impaired vaccine responses to generalized hypo-gammaglobulinemia (10, 12, 16). These may be incidental findings during diagnostic workup or lead to recurrent infections, particularly respiratory infections. Almost a third of patients develop lymphoma with the most common form being abdominal B cell non-Hodgkin lymphoma in both EBV+ and EBV− patients; prognosis has dramatically improved over the decades due to improved chemotherapy protocols.

Analyses of mutations have revealed deletions, splice site, nonsense, and missense changes in *SH2D1A*, but so far, there has not been a clear correlation between mutations and the severity of phenotypes identified (16, 26). Patients can progress from one phenotype to another, and different clinical features are seen within members of the same family. However, in some cases, second-site reversions of missense and nonsense mutations have been found, which were associated with restored CD8 cell function in a small fraction of cells and less severe phenotypes (27).

It is important to highlight that up to 35% of patients have no evidence of previous EBV infection; many of these patients are diagnosed based on family history (16, 28, 29). In EBV− patients, XLP1 is associated with higher rates of dysgammaglobulinemia (51.8 vs 37.2% for EBV+) and lymphoma [25 vs 19.6% for EBV+, see Table 2 from Ref. (16)]. However, EBV-negative boys with XLP1 can still develop HLH, although less frequently than those with EBV infection (21.4 vs 51% for EBV+) (16), and the trigger is unknown. Thus, XLP1 must be thought of as a disorder of immune dysregulation not only triggered by EBV. Nonetheless, there are no reports in the literature of a specific pathogen other than EBV being linked to HLH or other clinical features, arguing that XLP1 patients are specifically susceptible to EBV rather than other pathogens.

The overall mortality of the disease has reduced significantly since first reports from the registry, from 75 to 29% (16), largely due to improved chemotherapy and HSCT protocols, as well as improved monitoring and supportive care (which will be discussed later in this review). However, patients diagnosed at birth through family history still risk significant mortality despite close monitoring, highlighting the severity of this PID.

## Molecular Insight into Phenotypes of XLP1

Improved description of patient cohorts combined with the evolution of molecular techniques has widened our understanding of XLP1. However, equally important has been the investigation of the genetic cause of XLP1 and how lymphocyte development and function are affected by mutations of *SH2D1A* (4, 30).

### Cloning of the Gene and Studies of SAP-Mediated Signaling

In 1998, three groups identified a gene, now known as *SH2D1A*, that was mutated in patients with XLP. While two groups identified this gene by positional cloning (13, 31), a third group independently identified the same gene as encoding a small adaptor molecule that bound to the cytoplasmic tail of a T cell costimulatory protein, signaling lymphocyte activation molecule (SLAM) (14). Genetic mapping and sequencing revealed that this gene was mutated in samples from several XLP patients (14). The identification of *SH2D1A* has helped identify patients with the disease, but has also led to new insight into the signaling pathways regulated by SLAM family members and how they contribute to the pathophysiology of XLP1 (4, 5, 32).

The evaluation of the predicted gene product revealed that *SH2D1A* encodes a small (14 kDa/128 aa) protein that is now known as SAP, or SLAM-associated protein (14). Intriguingly, SAP consists almost entirely of a single Src Homology 2 (SH2) domain, a conserved protein interaction module that binds to phosphotyrosine-based motifs. SH2 domains are usually part of larger multi-domain proteins involved in signaling pathways, including adaptor molecules that contain multiple protein–protein and/or protein–lipid interaction domains and enzymes such as kinases and phosphatases that are regulated by intra- and intermolecular SH2-protein interactions (33). Further experiments demonstrated that the SH2 domain of SAP bound specific tyrosines on the intracellular tail of SLAM and related receptors (34, 35). However, these observations raised questions on how a single protein interaction domain could regulate signaling and how the disruption of SAP expression led to phenotypes associated with XLP1.

Although SAP was first identified by virtue of its association with SLAM, a costimulatory receptor that helps regulate interferon gamma cytokine production by T cells, SAP is now known to bind to a series of related receptors, the SLAM family, which include SLAM/CD150 (SLAMF1), LY9/CD229 (SLAMF3), 2B4/CD244 (SLAMF4), CD84 (SLAMF5), NTB-A/Ly108/CD352 (SLAMF6), and CRACC/CD319 (SLAMF7) (36). These receptors are encoded in a highly polymorphic gene cluster on human and mouse chromosome 1, variants of which have been associated with predispositions to autoimmunity (37). With the exception of 2B4, these receptors are self-ligands and are activated by homophilic interactions (30, 36). The SLAM family also has homology to the larger CD2 superfamily of immunoglobulin domain containing receptors, which include CD48/SLAMF2 (the ligand for 2B4/SLAMF4). SLAM receptors exhibit a broad expression on hematopoietic cells; however, several members are most highly expressed on B cells (38–41), a feature that likely contributes to some of the B cell-specific phenotypes of XLP1 (42). By contrast, although some B cell expression has also been reported (35, 43, 44), SAP is most highly expressed in T and NK cells and is therefore most likely to affect SLAM family function in these cells (14, 45). Several of the SLAM family members, including 2B4/SLAMF4, NTB-A/SLAMF6, and CRACC/SLAMF7, have been implicated as cytotoxic receptors in NK and CD8 cells (30).

**Figure 1:**
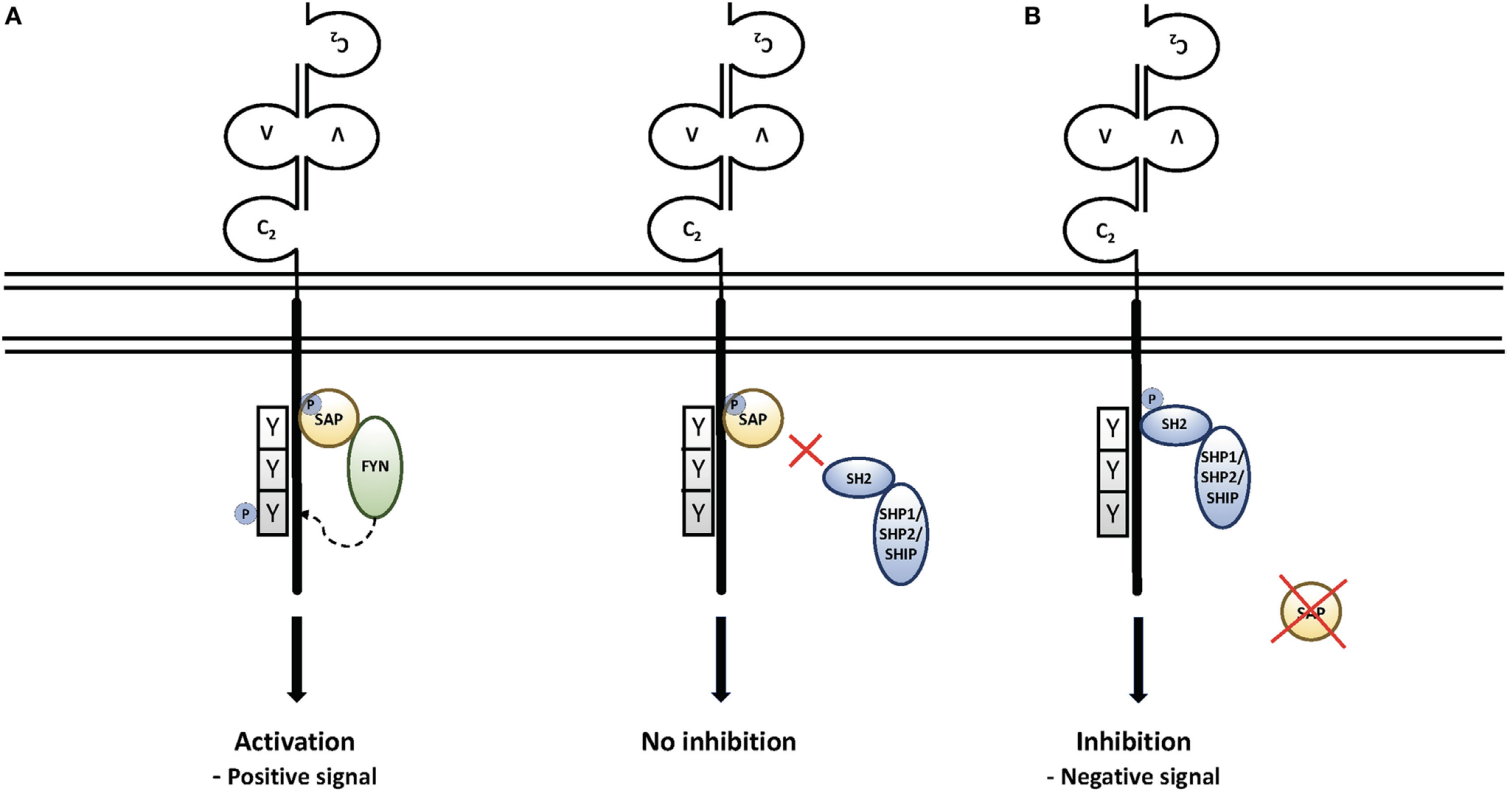
Signaling through the signaling lymphocyte activation molecule (SLAM) family receptors: **(A)** upon engagement, SLAM family members recruit the SAP SH2 domain to immunotyrosine switch (ITSM) motifs on their intracellular tails. This leads to the recruitment of Src family kinases, further phosphorylation of SLAM family receptors, and recruitment of downstream signaling molecules. Recruitment of SAP also prevents recruitment of the phosphatases SHP1, SHP2, and the lipid phosphatase SHIP (50). **(B)** In the absence of SAP, the engagement of SLAM family receptors leads to SH2-mediated recruitment of SHP1, SHP2, and SHIP, which are associated with strong inhibitory signals that affect T and NK cell function and cell death, as well as NKT cell development.

Extensive work on SAP-mediated signaling pathways provided evidence that SAP serves as a molecular switch allowing SLAM family members to act as either activating receptors in the presence of SAP or inhibitory receptors in the absence of SAP (Figure 1) (30, 35, 36, 46, 47). Thus, when SAP is present, it can recruit the FYN tyrosine kinase, leading to further tyrosine phosphorylation of SLAM family members (48–50) and interactions with other signaling molecules, including RasGAP, Shc, Dok1, and Dok2 in the case of SLAM (51) and Vav1 and c-Cbl in the case of 2B4 and Ly108 (41, 45, 52). Notably, Fyn deficiency can phenocopy some features of SAP deficiency including defects in 2B4-mediated killing (50). SAP has also been shown to inhibit diacylglycerol kinase-α (DGKα), a molecule that negatively affects TCR signaling (53). However, when SAP is not expressed, the same tyrosines on SLAM family members bind a number of strong inhibitory molecules, including the tyrosine phosphatases SHP1 and SHP2, as well as the lipid phosphatase SHIP (35, 41, 46, 47, 54). These inhibitory molecules essentially block aspects of T and NK cell activation, development and function when SLAM family members are engaged in the absence of SAP. Accordingly, the tyrosine-based motif that SAP recognizes has been coined an “ImmunoTyrosine Switch Motif” or ITSM (35).

Early data provided evidence that NK and CD8 cells from XLP patients exhibited defective killing of EBV-infected B cells (55, 56); this was linked to impaired killing *via* 2B4/SLAMF4 and NTB-A/SLAMF6 (57–60). Intriguingly, some data demonstrated that in the absence of SAP, 2B4/SLAMF4 prevented the killing of EBV-infected cells, providing further evidence that the SLAM family could act as inhibitory receptors (58). Combined with the biochemical evidence for the inhibitory function of SLAM family receptors, these results provided insight into why XLP1 patients have specific susceptibility to EBV infection. More recently, T cells from XLP1 patients have been found to exhibit defects in reactivation-induced cell death (RICD), resulting from inhibitory signaling from NTB-A/SLAMF6. This defect has been proposed to contribute to lymphoproliferation seen in XLP1 (61).

### Insight From Mouse Models

The generation and study of SAP-deficient mice (62–64) has provided insight into additional phenotypes associated with SAP deficiency, some of which have subsequently been confirmed in XLP1 patients. One of these is a lack of invariant NKT cells, a rare innate type of T lymphocyte that rapidly responds to infection and may be involved in tumor surveillance—this defect was recognized due to the connection with Fyn, which also affects NKT cell development in mice (65–67). Whether and how the absence of NKT cells contributes to manifestations of XLP1 remains less well understood, but it is now appreciated that XLP1 patients exhibit an absolute lack of iNKT cells, independent of EBV infection status. The critical role of SAP in iNKT development is supported by studies of *SH2D1A* carriers demonstrating random X-inactivation in T and B cells but non-random X-inactivation in iNKT cells, suggesting an absolute requirement of SAP for the development of this population (66).

Another major phenotype is the lack of long-term humoral (antibody) responses and memory B cells, which have been observed both in response to infection and to immunization in SAP-deficient mice (62, 64, 68–70). These phenotypes were T cell intrinsic and associated with impaired formation of germinal centers (GCs) (68, 70), the site where B cells undergo immunoglobulin gene class-switching and hypermutation in response to antigen in the context of contact-dependent signals from specific CD4 T helper lymphocytes, now known as follicular T helper (Tfh) cells (71). The GC is also the site where most memory B cells and long-lived plasma cells are derived. Subsequent evaluation of XLP1 patients revealed that they also lacked IgG^+^ memory (CD 27^+^) B cells, and an autopsy confirmed a lack of GCs in lymph nodes from an XLP1 patient (72). Interestingly, in addition to the well-documented dysgammaglobulinemia in XLP1 patients, evidence of impaired responses to protein immunization had been reported (73). However, the characterization of SAP-deficient mice has provided a clearer picture of the nature of these humoral defects (74).

Further insight into these phenotypes came from intravital microscopy in mice, which revealed that SAP-deficient T cells exhibited impaired adhesion to B cells, a defect that was confirmed using *in vitro* flow-based cell conjugation assays (75). This defect was relatively specific, as that adhesion to antigen-presenting dendritic cells was less affected. The B cell specificity correlated with very high levels of the expression of multiple SLAM family members including SLAMF6 (Ly108/NTB-A), SLAMF5 (CD84), and CD48 (the ligand for 2B4) on activated B cells (38, 40, 42). In the absence of SAP, some of these ligands trigger an inhibitory response in SAP-deficient T cells, preventing full activation by and adhesion to B cells, likely by affecting TCR-induced inside-out signaling to integrins (76, 77).

Consistent with these observations, SAP-deficient T cells are initially activated normally by antigen-presenting dendritic cells in response to immunization and infection, but fail to form mature Tfh cells, a process now recognized to require B cell interactions (75, 78, 79). Indeed, insight into the critical role of Tfh cells in humoral immunity has been greatly advanced by studies of SAP-deficient mice. Such findings further suggested that defective adhesion to B cells was likely to contribute to the inability of SAP-deficient T cells to provide contact-dependent help for GC generation and long-term humoral immunity and thus the dysgammaglobulinemias seen in XLP1 (42, 75).

**Figure 2:**
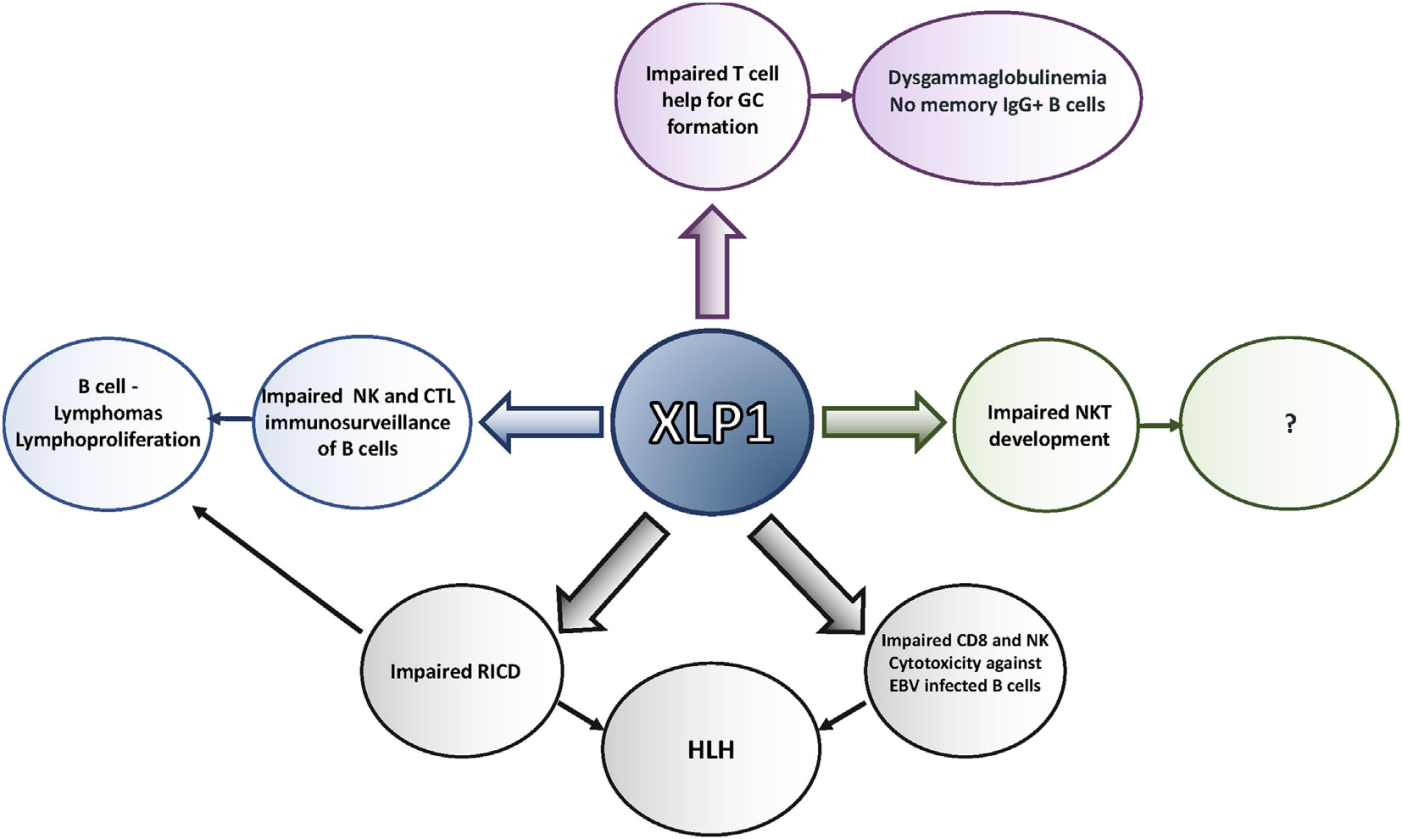
Defects seen in X-linked lymphoproliferative disease (XLP)1. XLP1 is associated with multiple T and NK cellular defects, many of which may result from impaired interactions with B cells and other cells that express high levels of signaling lymphocyte activation molecule (SLAM) family members. These defects contribute to the phenotypes observed in the disease, including the inability to clear Epstein–Barr virus (EBV), lymphoproliferation and lymphoma, hemophagocytic lymphohistiocytosis (HLH), and antibody defects.

Moreover, the observation of defective interactions with B cells has provided mechanistic insight into other phenotypes of XLP1, many of which share a common feature of B cell involvement (Figure 2). SAP-deficient CD8 cells exhibit defective adhesion to and killing of activated B cell targets (39–41, 80), especially EBV-transformed cells, which express high levels of certain SLAM family members and CD48. Thus, the sensitivity to EBV may occur because EBV primarily infects B cells. Impaired immunosurveillance of B cell malignancies may contribute to the increased incidence of lymphomas, even in the absence of EBV infection (16, 42). Since defective CD8 and NK cell cytolysis have been linked to HLH, defects in killing EBV-infected B cells may trigger this phenotype as well (81), although the exact mechanism by which HLH develops in this population is yet to be elucidated. Moreover, since other hematopoietic cells also express SLAM family members, defects may be extended to cytolysis of other hematopoietic targets; defects in NK cell cytolysis of multiple hematopoietic cell tumor lines that express SLAM family members have been observed (82). Nonetheless, it is also of note that NK cells deficient in SAP can kill non-hematopoietic cell targets better, perhaps accounting for the lack of increases in other types of cancer in XLP1 (83). Finally, the absence of NKT cells may result from impaired interactions between lymphocytes, as that NKT cells are not selected on the thymic stroma, but rather on double-positive thymocytes that express high levels of SLAM family members (84).

It is of note that the effects of SLAM family receptor mutations for the most part do not phenocopy those of SAP deficiency (85). Moreover, although polymorphisms of SLAM family members are associated with autoimmunity in lupus-prone mouse strains and humans (37), and with alterations in NKT cell numbers in NOD (non-obese diabetic) mice (86), to date, there have been no reports of immunodeficiency or EBV susceptibility associated with mutations of other SLAM family members in humans (36). Instead, many of the phenotypes of SAP deficiency appear to be related to inhibitory signals generated by SLAM family members in the absence of SAP (39, 76, 85), which are most strongly triggered by B cell interactions, either as targets (for cytolysis) or as antigen presentation (GC formation). Notably, unlike positive signaling, these inhibitory signals would not be expected to rely on the ability of SAP to recruit Fyn. Indeed, several phenotypes associated with SAP deficiency can be rescued by the expression of a mutant of SAP that cannot recruit Fyn but can still block the recruitment of inhibitory molecules. These include humoral defects, NK cell killing, and NK cell education (54, 70, 78, 83), although some phenotypes such as 2B4-mediated killing and NKT cell development may result from defects in both positive and negative signals since Fyn deficiency also impairs these processes (50, 87). Importantly, unlike positive signaling, these negative signals require the presence of SLAM family members to recruit phosphatases and manifest their inhibitory function (Figure 1). Thus, inhibiting interactions of the SLAM family members 2B4/SLAMF4 and NTB-A/SLAMF6 with their ligands actually improves cytolysis of B cells by SAP-deficient CD8 cells and NK cells (39, 41, 58). These observations suggest the intriguing possibility that blocking antibodies to SLAM family receptors might ameliorate some of the clinical manifestations of this disorder, raising the possibility of tailored SLAM family-based pharmacological approaches to XLP1 (see below). Support for this hypothesis can be found in murine genetic studies where mutations disrupting the expression of Ly108/SLAMF6 improved both the GC defect and NKT development in SAP-deficient mice (76).

## Current Treatment and Management Options for XLP1

Given the severe morbidity and high rates of mortality in XLP1, it is strongly recommended that genetic screening and counseling be carried out in families with a history of XLP1 (5). Diagnosis is confirmed using flow cytometric analyses of SAP expression (88) followed by Sanger sequencing of the *SH2D1A* gene. Immunological status is assessed with focus on immunoglobulin levels and response to vaccines.

Currently, the only definitive treatment available for XLP1 patients is allogeneic HSCT (16). However, depending on clinical features, less aggressive treatments may be adopted, particularly if a suitable donor for transplant is not available. As many patients do not present with all symptoms simultaneously or at varying severity, there are a number of treatment options that target specific clinical phenotypes.

### Treatment Approaches

Treatment of XLP1 is tailored to particular clinical symptoms and supportive care. However, close monitoring (e.g., of EBV viral loads) is important in this patient cohort to allow the prevention of recurrent infections, organ damage such as bronchiectasis, and permit early treatment of EBV infection and more serious complications. If there is evidence of EBV-driven disease, including HLH, treatment with a monoclonal anti-CD20 antibody (rituximab) can be used to deplete the B cell population harboring the virus (89). This approach is effective at reducing and often clearing the viremia but risks the effects of B cell depletion, including exacerbating long-term hypo-gammaglobulinemia. Antiviral agents are poorly effective against EBV but acyclovir has been used in some circumstances. Infection of T cells with EBV is also seen in XLP1 patients (unpublished data) and the use of rituximab in this situation may not be helpful.

Hemophagocytic lymphohistiocytosis is treated according to standardized protocols (HLH 94 and 2004) based on the use of dexamethasone, etoposide, and cyclosporin with the addition of intrathecal methotrexate and steroids if there is neurological involvement (90, 91). This is a highly suppressive regime and can be associated with significant toxicity. The protocol follows different stages, starting with an intense period of treatment initially, with reducing doses of steroids and frequency of etoposide over time if a response is seen. Re-intensification of therapy is occasionally required. This protocol aims to achieve remission of HLH, usually prior to moving swiftly to HSCT, but the mortality associated with this presentation is still over 60% (16). Other immunosuppressive agents have been used to control HLH, either in combination with steroids or as rescue therapy, including ATG (anti-thymocyte globulin) in combination with etoposide in the HIT (hybrid ImmunoTherapy)-HLH trial (NCT01104025), or Alemtuzumab (Campath/anti CD52 antibody). In addition, newer biologics are now available, and some are being tested in HLH including Toculizumab (anti-IL6R antibody). An anti-interferon gamma monoclonal antibody (Novimmune NI-0501) is now in trial in the USA and Europe with results eagerly awaited. The JAK1/2 inhibitor Ruxolitinib has shown promise in preclinical murine studies and is now also moving toward the clinic (92).

These more targeted therapies could offer an improved toxicity profile, which may be extremely beneficial to help transition patients rapidly to HSCT with as little organ damage and infectious complications as possible and thereby afford better outcomes post transplant. Lymphoma is also treated according to standardized protocols, and again mortality associated with this presentation has reduced over the years.

Patients with dysgammaglobulinemia or recurrent infections may benefit from immunoglobulin replacement therapy which can be delivered *via* intravenous route every few weeks, or subcutaneously every week, which is usually performed at home. Other manifestations of dysregulation such as aplastic anemia or vasculitis may respond to steroid therapy or other immunosuppressive agents.

### Stem Cell Transplantation

Bone marrow (BM) or HSC transplantation (which includes the transfer of BM, mobilized CD34^+^ cells from peripheral blood or umbilical cord-derived CD34^+^ cells) is currently the only definitive treatment for XLP1; survival for untransplanted patients is below 20% (16). However, success is dependent on the availability of an appropriate donor who is human leukocyte antigen matched (16). There are a number of factors to consider prior to HSCT, including the disease status, previous treatments, and the type of pre-conditioning regimen. An EBV-positive donor is preferred in patients with EBV-driven disease.

Several studies have evaluated the clinical outcomes of patients undergoing HSCT using either myeloablative-conditioning regimens or reduced-intensity-conditioning (RIC) regimens (16, 93, 94). These studies revealed similar overall patient survival rates post transplantation between RIC and myeloablative protocols, with both averaging ~80% (16, 94). However, success rates drop depending on the presence of active HLH at the time of transplant (falling to 50%) and in the context of a mismatched donor (16). From this large cohort, all patients who died post HSCT had evidence of HLH.

Thus, although the survival in XLP1 has improved significantly over time, it remains a potentially fatal condition. The decision to undertake an HSCT in an asymptomatic patient requires intensive discussion with the family to understand both risks and benefits, especially when a mismatched donor is the available choice. However, many families are faced with severe complications at presentation, such as HLH or lymphoma, which necessitate a rapid move to HSCT.

### Potential Future Therapies

#### SLAM Family Inhibitors

In the absence of SAP, the recruitment of phosphatases and other inhibitory signaling molecules convert SLAM family members into inhibitory receptors (4). This is particularly relevant for SLAMF4/2B4/CD244 and SLAMF6/NTB-A, which strongly inhibit CD8 and NK cell killing of B cell targets in the absence of SAP. Preventing SLAMF4/2B4 and/or SLAMF6/NTB-A engagement, either through genetic knockouts of these receptors in mice or through the use of blocking antibodies with human cells, can rescue phenotypes associated with SAP deficiency, including the defective killing of B cell targets, the absence of GC formation, defective NKT cell development, NK cell education, and impaired RICD (39, 41, 61, 76, 83). Limiting the homophilic interactions of SLAM family receptors (or in the case of SLAMF4/2B4, interactions with its ligand, CD48) in XLP1 patients may therefore prevent lymphoproliferation and other phenotypes of XLP1. *In vitro* experiments have provided evidence that blocking antibodies against CD48 and NTB-A rescue killing of EBV-infected targets, supporting the concept of humanized blocking antibodies as a potentially useful therapy (39). Alternatively, peptide(s) or small molecules with a high affinity for the different SLAM receptors might block SLAM family interactions and the initiation of a negative signal.

Other potential therapeutic approaches include the use of small molecule inhibitors of signaling pathways affected by SAP and SLAM family members. The inhibition of SHP1/SHP2 rescued cytolysis of B cell targets *in vitro* using murine cells (41). Other data suggest that the inhibition of DGKα, another negative regulator of T cell activation that is affected by SAP, can rescue certain phenotypes associated with SAP deficiency, including RICD and hyperproliferative responses to lymphochoriomeningitis virus in mice (53, 95). However, none of these approaches are curative, and toxicity may be a major issue, particularly for long-term treatment.

#### Gene Therapy

Over the last few years, there have been great strides developing effective and safe hematopoietic stem cell gene therapy as a viable alternative to BM transplantation for a number of PIDs. Gene therapy also offers the advantages of reduced toxicity from conditioning as, in general, less chemotherapy is required and the use of autologous cells removes the risk of graft versus host disease which causes significant morbidity and mortality post HSCT (96, 97). Although several first-generation gene therapy trials were marred by the integration of gammaretroviral vectors near proto-oncogenes leading to leukemia and myelodysplasia, newer self-inactivating (16) retroviruses and lentiviruses have been developed that use internal mammalian promoters to drive transgene expression. Numerous clinical trials are underway using these later generation vectors, and no insertional events have been reported to date.

A proof of concept for gene therapy for XLP1 was established using such a second-generation lentiviral vector containing the human elongation factor 1 alpha promoter to drive codon-optimized SAP gene expression (98). This study utilized a SAP-deficient murine model into which gene-corrected hematopoietic progenitor cells were infused following lethal irradiation. The transfer of gene-corrected cells led to the restoration of NK and CD8 T cell cytotoxicity, NKT development, as well as GC formation and function upon immunological challenge. However, although no adverse effects of SAP expression at the stem cell level were seen in these studies, SAP is a tightly regulated signaling protein that is predominately expressed in T cells (14, 45), and the use of a ubiquitous human promoter that can drive expression in all hematopoietic cells may not be optimal.

An alternative approach to more directly address the T cell-dependent clinical manifestations of XLP1 is to develop a therapeutic strategy using gene-corrected autologous patient T cells. Murine studies utilizing gene-modified T cell transfers into *Sh2d1a*^−/y^ mice demonstrated the correction of Tfh cell function, the restoration of GCs, and the improvement in baseline immunoglobulin levels (Panchal et al., in press). In addition, the correction of CD8^+^ T cell function was shown using an *in vivo* tumor model. These data provide a strong case that adoptive T gene therapy may be a useful therapeutic option.

#### Gene Editing

Along with developments in gene therapy, the latter part of this decade has seen great advancements in the use of gene-editing platforms for therapeutic benefits (96, 99–101). Zinc finger nucleases have been established to be effective in eliminating CCR5 expression on T cells from HIV-infected individuals in order to prevent viral spread (102, 103). Transcription activator-like effector nucleases and CRISPR/Cas9 nuclease systems have been used for TCR knockdowns as part of CAR-T cell therapy, to produce an “off the shelf” donor T cell product for the treatment of CD19^+^ B cell leukemias (104, 105). Gene-editing platforms hold great promise for the effective correction of endogenous genes using corrected DNA copies as donor templates, utilizing the cell’s own DNA repair machinery. This approach may be particularly beneficial for monogenic diseases such as XLP1 that can present with point mutations in the gene. Gene editing also offers a resolution to the issue of gene regulation and the risk of overexpression in anomalous hematopoietic compartments and could significantly improve the safety profile of genetically engineered cellular therapy (100, 106). However, this type of therapy needs to be custom-designed to repair the genetic defect of each patient and may not be useful for patients with gene deletions. Potential off-target effects also need to be carefully evaluated. Nonetheless, such approaches hold high potential for the treatment of PIDs.

## Summary

Over the last 30 years, the outcome for patients with XLP1 has significantly improved, mainly due to improvements in the treatment of clinical manifestations such as HLH and lymphoma. Survival post HSCT has also improved, but mortality associated with active disease at the time of transplant and mismatched donor settings remains significant. As our understanding of the molecular and cellular pathology in XLP1 continues to expand, novel treatments, including gene therapy, will continue to be developed, hopefully leading to even greater improved outcomes for patients with this devastating disease.

## Author Contributions

NP, CB, JC, and PS all contributed to the writing and editing of this manuscript. NP and PS generated the figures.

## Conflict of Interest Statement

The authors declare that the research was conducted in the absence of any commercial or financial relationships that could be construed as a potential conflict of interest.
